# CRISPR-based gene editing technology and its application in microbial engineering

**DOI:** 10.1016/j.engmic.2023.100101

**Published:** 2023-06-20

**Authors:** Junwei Wei, Yingjun Li

**Affiliations:** State Key Laboratory of Agricultural Microbiology and College of Life Science and Technology, Huazhong Agricultural University, Wuhan 430070, China

**Keywords:** CRISPR-Cas system, Gene editing, Industrial microorganisms, Probiotics

## Abstract

Gene editing technology involves the modification of a specific target gene to obtain a new function or phenotype. Recent advances in clustered regularly interspaced short palindromic repeats (CRISPR)-Cas-mediated technologies have provided an efficient tool for genetic engineering of cells and organisms. Here, we review the three emerging gene editing tools (ZFNs, TALENs, and CRISPR-Cas) and briefly introduce the principle, classification, and mechanisms of the CRISPR-Cas systems. Strategies for gene editing based on endogenous and exogenous CRISPR-Cas systems, as well as the novel base editor (BE), prime editor (PE), and CRISPR-associated transposase (CAST) technologies, are described in detail. In addition, we summarize recent developments in the application of CRISPR-based gene editing tools for industrial microorganism and probiotics modifications. Finally, the potential challenges and future perspectives of CRISPR-based gene editing tools are discussed.

## Graphical abstract

**Figure:**
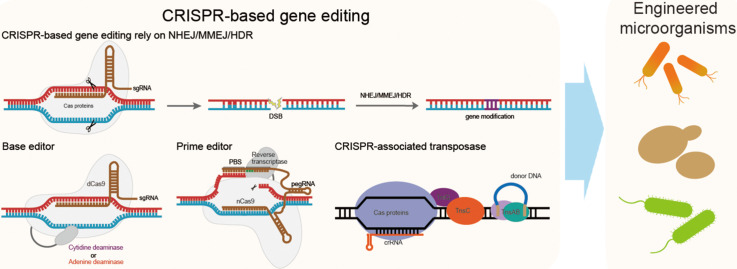

## 1 Introduction

The rapid development of life sciences and biotechnology has led us from the "reading" stage of genetic information to the post-genomic era, where "rewriting" and even "new design" of the genome is becoming a reality. With continuous exploration and iteration, clustered regularly interspaced short palindromic repeats (CRISPR)-based gene editing tools have become mainstream, and their simplicity and efficiency have greatly advanced the development of many life science fields. The history of gene editing technology can be traced back to 1953, when Watson and Crick proposed the double helix structure of the DNA molecule [1]. By this time, scientists had realized the decisive role of DNA in biological traits and phenotypes, and this great discovery also inspired the idea of artificial modification of DNA. Gene editing technology has gradually developed from this background.

**Fig. 1:**
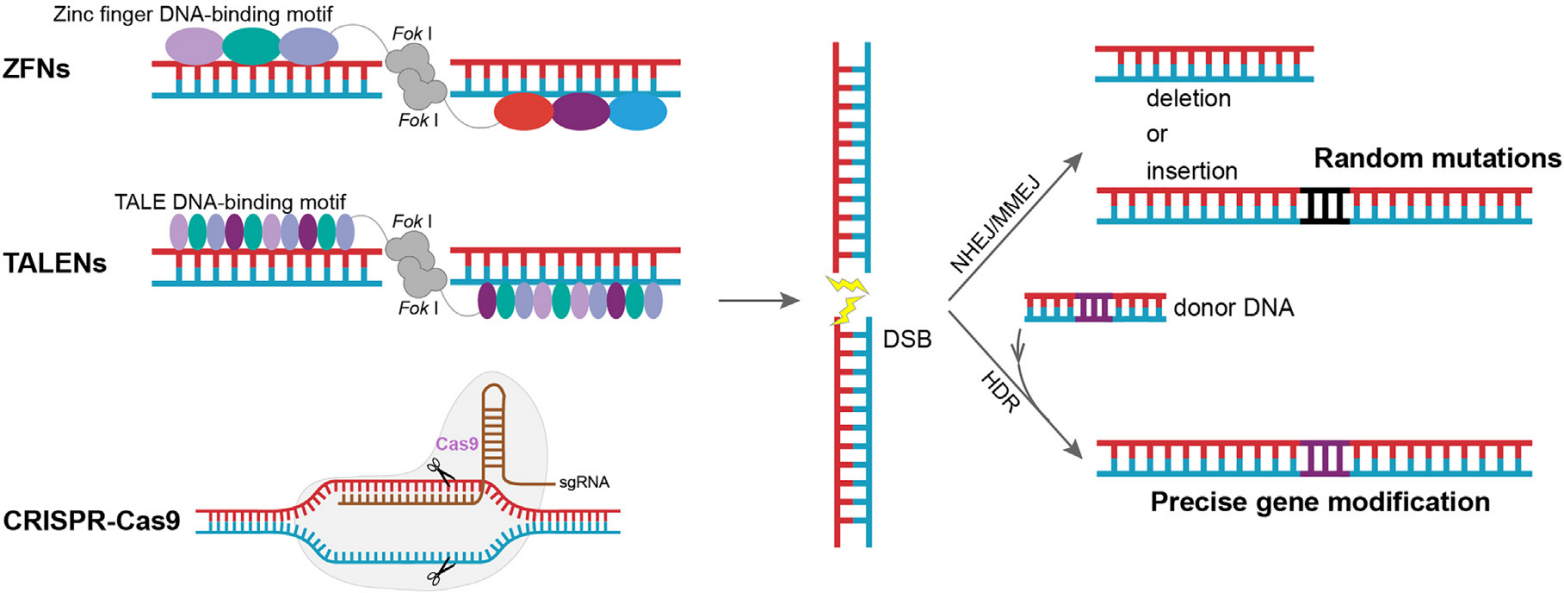
Comparison of gene editing methods based on ZFNs, TALENs, and CRISPR-Cas9 technologies. Both ZFNs and TALENs are engineered nucleases, and their *Fok* I nucleases are fused with codon-specific and nucleotide-specific recognition modules, respectively. After the recognition module binds to the target site, *Fok* I nucleases are activated after dimerization. CRISPR-Cas9, however, is an RNA-guided nuclease that is activated after the guide RNA is base-paired with target sites. These systems all cause DNA double-strand breaks (DSBs) near the target site, introducing gene editing through subsequent repair pathways such as NHEJ, MMEJ, or HDR. The latest CRISPR-based gene editing tools that do not rely on DSBs are described in a later section.

Early gene editing techniques relied on spontaneous homologous recombination (HR) pathways in organisms, where mutations were generated by exchange and rearrangement between target sites and provided homologous donors [2]. However, the occurrence frequency of HR is extremely low, with only ∼10^−6^ to 10^−9^ in eukaryotes [3]. Subsequently, it was shown that the HR occurrence frequency could be increased by the activation of host damage repair pathways through DNA double-strand breaks (DSBs) [4,5]. As a result, a series of nuclease-based gene editing tools have emerged. In brief, programmable nucleases cause DSBs at specific sites in the genome, then mutations are introduced through subsequent damage repair pathways, including non-homologous end joining (NHEJ), microhomology-mediated end joining (MMEJ), and homology-directed repair (HDR) [6]. Among these nuclease-based gene editing techniques, zinc finger nucleases (ZFNs), transcription activator-like effector nucleases (TALENs), and CRISPR-based technologies are the most widely used and well-known (Fig. 1). ZFNs and TALENs are very similar, as both are engineered nucleases. Zinc finger protein (ZFP) or transcription activator-like effectors (TALEs) are responsible for target recognition and fused *Fok* I endonuclease is responsible for DNA cleavage. The emergence of ZFNs was a great advancement in gene editing technology, but it has significant limitations, such as high cost, complicated design, high off-target rate, and difficulty in achieving multi-target editing [7], [8], [9]. TALENs is an improved technology that was based on ZFNs. For the same target sites, the cleavage efficiencies of TALENs and ZFNs were found to be not very different, but TALENs have lower intracellular toxicity [8,10]. TALENs are also easier to design than ZFNs, allowing them to theoretically be designed and constructed for any target sequence [7]. However, each base of the target sequence requires a corresponding TALE recognition module, so TALENs have a large associated construction workload. ZFNs and TALENs can improve gene knockout efficiency, but they both rely on engineered proteins for specific recognition of DNA, resulting in a need for new proteins to be designed for different target sites. Cumbersome operation and high technical threshold are the main obstacles limiting their application in gene editing [11]. Subsequently, the advent of the CRISPR-Cas system, which is based on short RNAs for target site identification, completely compensated for the shortcomings of these various earlier gene editing techniques. Compared with the complex protein engineering associated with ZFNs and TALENs, the CRISPR-Cas system enables targeting of different sequences by simply changing the guide RNA. In addition, base-pairing-based recognition of target sites has higher specificity compared with protein-based recognition [12].

Recently, CRISPR-based gene editing tools have been widely used in all three domains of life (eukaryotes, prokaryotes, and archaea) [13], [14], [15]. Additionally, several derivative technologies developed based on the CRISPR-Cas system have also all demonstrated powerful gene manipulation capabilities, such as gene transcriptional regulation tools, CRISPR interference (CRISPRi), and CRISPR activation (CRISPRa) [16], [17], [18], as well as novel gene editing tools that do not rely on DSBs, base editors (BEs) [19], [20], [21], [22], [23], prime editors (PEs) [24], [25], [26], [27], and CRISPR-associated transposases (CASTs) [28]. Genetic improvement of microorganisms using gene editing technologies is of great significance for the utilization of microbial resources. Among them, genetic improvement of industrial chassis microorganisms and probiotics are two important areas of research. The development of industrial chassis microorganisms can provide excellent cell factories that can contribute to the realization of environmental protection and a sustainable economy. Moreover, probiotics have long been widely used in pharmaceuticals and food, and their modification by gene editing will further improve their probiotic functions, which has good prospects for industrialization. Previous reviews of CRISPR-Cas technology have focused on the detailed working mechanisms of different types of CRISPR-Cas systems and their applications in some higher organisms, such as plants and animals [6,[29], [30], [31]]. Here, our review focuses on summarizing traditional and emerging CRISPR-based gene editing technologies and their applications for microbial modification, including industrial chassis microbes and probiotics. In this review, we describe the classification and mechanism of the CRISPR-Cas system and introduce various gene editing techniques that are based on this system. We also summarize the application progress of CRISPR-based gene editing tools in industrial microorganisms as well as probiotics. The limitations of the current CRISPR-based gene editing tools and prospects are also discussed.

## 2 Definition and principle of the CRISPR-Cas system

The CRISPR-Cas system is an acquired immune system in prokaryotes to fight against foreign genetic elements. It is widely present in about 50% of bacteria and 90% of archaea species that have been sequenced [32,33]. The CRISPR-Cas system is composed of two genetic elements, CRISPR arrays and a series of *cas* genes encoding Cas proteins. The CRISPR array starts with an AT-rich leader sequence that generally acts as a promoter and recognition site for new spacer sequence integration [30]. Identical repeat sequences and specific spacer sequences are alternately arranged after the leader sequence. Repeat sequences of homogeneous CRISPR arrays are highly conserved, while those of different types of CRISPR arrays are different in sequence and structure [32]. The CRISPR array is flanked by diverse *cas* genes that encode certain Cas proteins with endonuclease domains, RNA and DNA binding domains, and domains involved in transcriptional regulation [34], [35], [36].

**Fig. 2:**
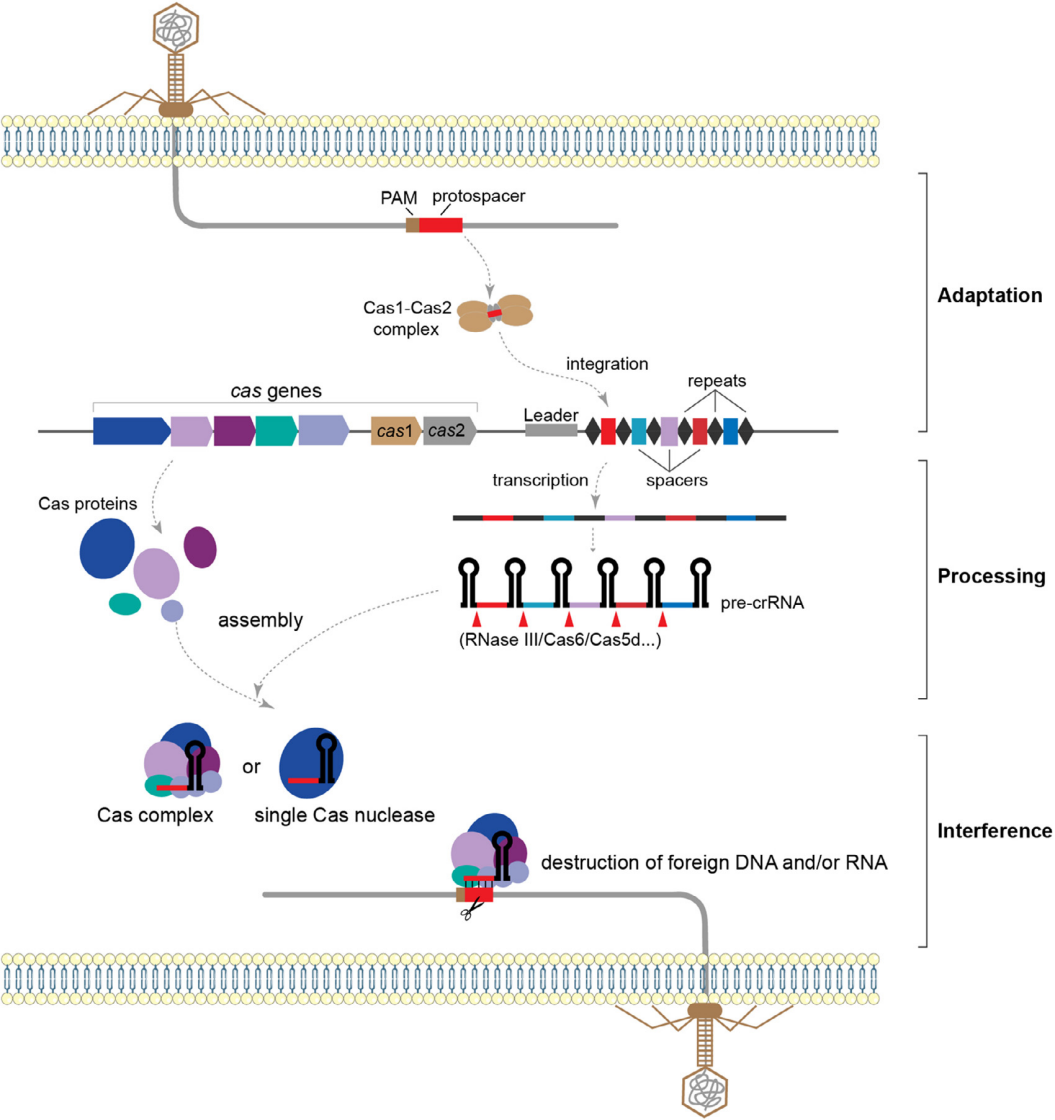
The three stages (adaptation, processing, and interference) of CRISPR-Cas immunity against invading genetic elements. During the adaptation stage, short fragments of foreign nucleic acids are integrated into the CRISPR array to form an immune memory. The CRISPR array is then transcribed into pre-crRNA, which is processed into mature crRNA by a related ribonuclease. In the final stage of target interference, mature crRNAs guide the CRISPR-Cas effector complex to cleave foreign nucleic acids through complementary base-pairing between the crRNA and target nucleic acid.

In general, the immune process of the CRISPR-Cas system mainly includes three stages: adaptation, processing, and interference (Fig. 2). In adaptation, also known as spacer acquisition, new spacers are captured from invading nucleic acids and integrated into the CRISPR array immediately following the leader sequence as a new spacer. Normally, a stable complex consisting of two Cas1 dimers and a single Cas2 dimer is essential for this stage [37], and part of CRISPR-Cas systems also require other auxiliary proteins, such as Cas4 and reverse transcriptase, to be involved in this process [38]. The second stage is the maturation of CRISPR RNA (crRNA). The entire CRISPR array is first transcribed into a long precursor crRNA (pre-crRNA), driven by promoter elements embedded in the leader sequence. The ribonucleases involved in pre-crRNA maturation include Cas nucleases, such as Cas6, Cas5d, and Cas12, and certain housekeeping non-Cas nucleases, such as RNase III, RNase E, and PNPase [30,[39], [40], [41]]. The mature crRNA then binds to effector proteins or multi-subunit effector complexes to form a ribonucleoprotein complex (RNP). In the final stage of target interference, the RNP recognizes and cleaves the invading DNA or RNA through complementary base-pairing between the crRNA and target nucleic acid [30,34].

## 3 Classification and interference mechanism of the CRISPR-Cas system

**Fig. 3:**
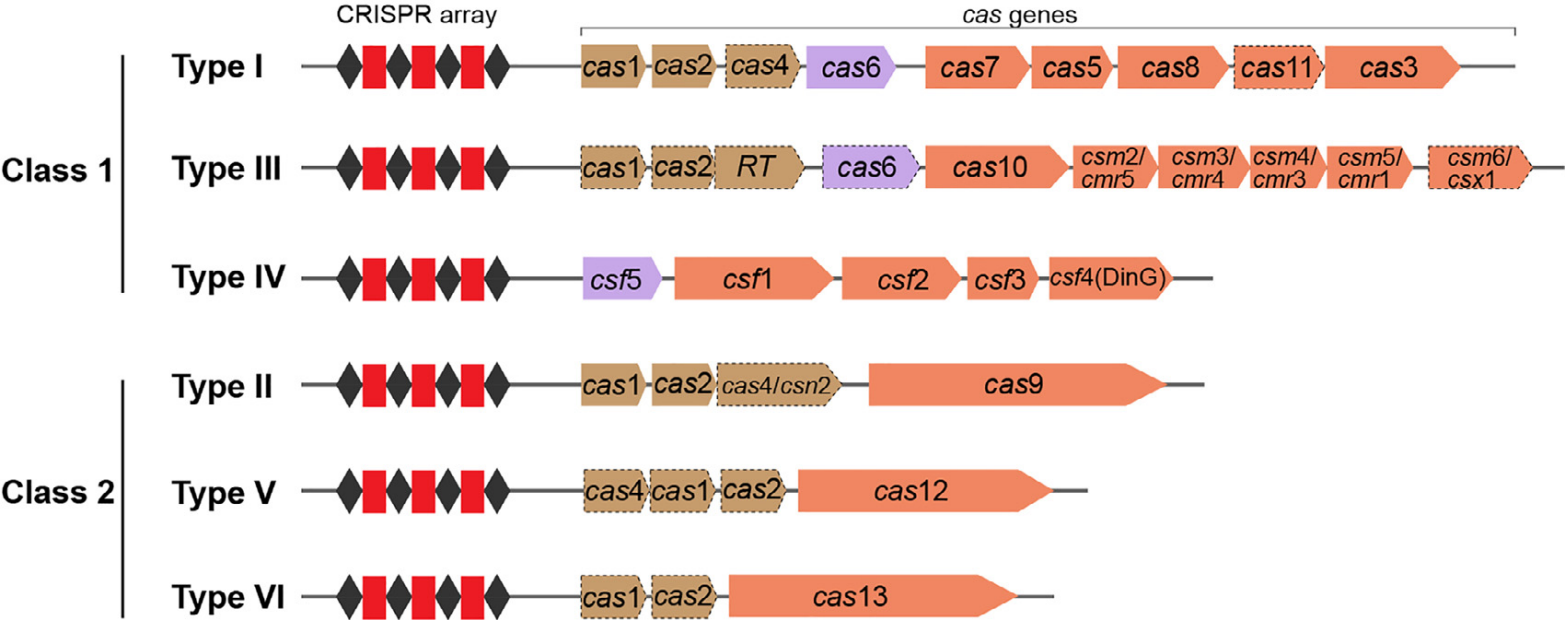
Schematic diagram of the classification and architecture of different types of CRISPR-Cas systems. The CRISPR-Cas system is highly diverse and is currently divided into two broad categories: class 1 and class 2. Class 1 systems encode multi-subunit effector complexes, while class 2 systems encode single-subunit effectors. Different types of systems encode specific Cas proteins involved in adaptation, processing, and interference. Genes that may be missing in some subtypes are indicated by dashed outlines. Genes encoding adaptation proteins are shown in brown. Genes encoding proteins involved in pre-crRNA processing are shown in purple. Genes encoding proteins involved in target interference are shown in brick red. The staggered diamonds and rectangles represent repeats and spacers, respectively, which make up the CRISPR array.

Based on the architectural composition of Cas proteins and effector complexes, the CRISPR-Cas system is currently divided into two classes (class 1 and class 2), including six types (types I to VI). Each type possesses its signature *cas* gene and is subdivided into 33 subtypes and several variants [42,43]. The class 1 CRISPR-Cas systems include types I, III, and IV, which use a complex of multiple Cas protein subunits to exert immune defense. The class 2 systems include types II, V, and VI, which use a single Cas protein effector to fight against invaders (Fig. 3, Table 1) [43]. Almost all identified CRISPR-Cas systems (except type III) require the recognition of a short sequence located in the 5′ flanking region of the protospacer (termed the protospacer adjacent motif, PAM) to mediate adaptation and interference [30,44,45]. In class 1 CRISPR-Cas systems, type I systems use a multi-subunit RNP, called CRISPR-associated complex for antiviral defense (Cascade), for target DNA recognition and cleavage [34]. Type I systems typically contain one Cas5 subunit, one Cas6 subunit, several Cas7 subunits, and the characteristic protein Cas3. Almost all pre-crRNAs of type I systems are processed by Cas6 family proteins, except for the I-C subtype system, which uses Cas5d [46,47]. When the type I system works, Cascade first uses Cse1 (Cas8) to search for the PAM sequence on the target DNA [48]. After identifying the specific PAM sequence, Cascade induces target DNA strand separation and crRNA pairing with the target DNA to form an R-loop for Cascade immobilization [49], [50], [51]. Afterwards, Cascade undergoes a conformational shift and recruits Cas3 through the Cse1 subunit of the complex. Cas3 possesses both helicase and nuclease activities and performs 3′ to 5′ degradation of the non-target chain [30,52].

**Table 1:** The six types of CRISPR-Cas systems.

Class	Type	Spacer acquisition	pre-crRNA processing	crRNP	Signature protein	Target nucleic acid
1	I	Cas1, Cas2, Cas4	Cas6(Cas5d for I-C)	Cascade	Cas3	DNA
	III	Cas1, Cas2, RT	Cas6/RNase E/ RNase H	Csm/Cmr	Cas10	DNA, RNA
	IV	unknown	Csf5	Csf	Csf1	DNA
2	II	Cas1, Cas2, Cas4/Csn2	RNase III, Cas9	Cas9	Cas9	DNA
	V	Cas1, Cas2, Cas4	Cas12	Cas12	Cas12	DNA
	VI	Cas1, Cas2	Cas13	Cas13	Cas13	RNA

The composition of the type III system is similar to that of the type I system, but its signature protein is Cas10. The type III system is more complex and can degrade both DNA and RNA [53], [54], [55]. During the interference phase, the type III system specifically targets RNA substrates. In addition, base pairing between crRNA and nascent RNA transcripts can activate multiple activities of type III systems. First, the histidine-aspartate (HD) nuclease domain of Cas10 is activated for non-specific cleavage of single-stranded DNA [56]. Simultaneously, the Palm domain of Cas10 is activated as a cyclic oligonucleotide (cOA) synthetase to convert cytoplasmic ATP into cOAs [57,58], which then act as second messengers to activate CRISPR-associated Rossman fold (CARF) domain-containing ribonucleases (Csm6/Csx1 families) [57]. The CARF structural domain of Csm6/Csx1 is allosteric upon sensing cOAs to activate its higher eukaryotes and prokaryotes nucleotide-binding (HEPN) domain, which is capable of non-specific RNA degradation [57,59]. This leads to an overall depletion of host and exogenous transcripts, resulting in a stagnation of host growth [60] and preventing the propagation of invaders in a manner similar to abortion infection (Abi) [61]. Type IV systems, which differ significantly from type I and III systems, are often located on plasmids and lack the Cas1 gene, Cas2 gene, and nuclease gene (Cas3 or Cas10) for interference, but encode Cas5 (Csf3), Cas7 (Csf2), and the characteristic protein Csf1 [62]. However, functional research on the type IV system is still relatively lacking. The few published studies have only described its crRNA maturation and PAM preference, while its obvious target interference with DNA nuclease activity has not been observed [63,64].

The class 2 systems include type II, V, and VI CRISPR-Cas systems, which prominently use a single Cas protein to defend against exogenous invaders. The signature proteins of type II, V, and VI CRISPR-Cas systems are Cas9, Cas12, and Cas13, respectively. For crRNA maturation, the crRNA processing protein of the class 2 systems is generally the target interference protein itself. For target interference, Cas9 and Cas12 are both RNA-guided DNA endonucleases. Cas9 can recognize multiple PAM sequences on the non-target strand to produce a blunt DNA DSB [65,66], while Cas12a recognizes T-rich PAM sequences on both DNA strands to produce a sticky DNA DSB [67,68]. Unlike Cas9 and Cas12, Cas13 of the type VI systems lack a DNase domain and instead contains two conserved HEPN domains [69]. The specific target substrate of type VI systems is single-stranded RNA (ssRNA), while base pairing of target RNA with crRNA can also activate non-specific RNase activity of Cas13, similar to the cleavage activity of Cas10 on single-stranded DNA (ssDNA) in the type III system [70].

## 4 Gene editing based on the CRISPR-Cas system

The ability to regulate and edit genetic information is essential for studying gene function, revealing biological mechanisms, and utilizing biological resources. The current mature gene editing strategies are essentially based on programmable nucleases. Among them, CRISPR-based gene editing tools are the most mature and widely used. As mentioned above, the main difficulty of ZFNs and TALENs for gene editing is that different target-binding proteins need to be designed and expressed for different editing sites. These are difficult to design, time-consuming, and laborious [11]. In contrast, the CRISPR-Cas system involves an RNA-guide nuclease that can be designed for different target sites by simply changing the guide RNA sequence. This process is simple and efficient, and has a higher specificity based on complementary base pairing [12]. These various advantages have resulted in CRISPR-based gene editing tools quickly and completely replacing ZFNs and TALENs as the next generation of gene editing technology. Through continuous iteration and development, two generations of CRISPR-based editing tools have been developed. First-generation technologies enable gene editing by introducing DSBs at specific sites and rely on DNA damage repair pathways. The emerging second-generation technologies use Cas proteins with abolished nuclease activity and therefore do not induce DSBs, enabling precise gene editing by fusing other functional proteins to Cas proteins.

### 4.1 CRISPR-based gene editing dependent on DSBs

**Fig. 4:**
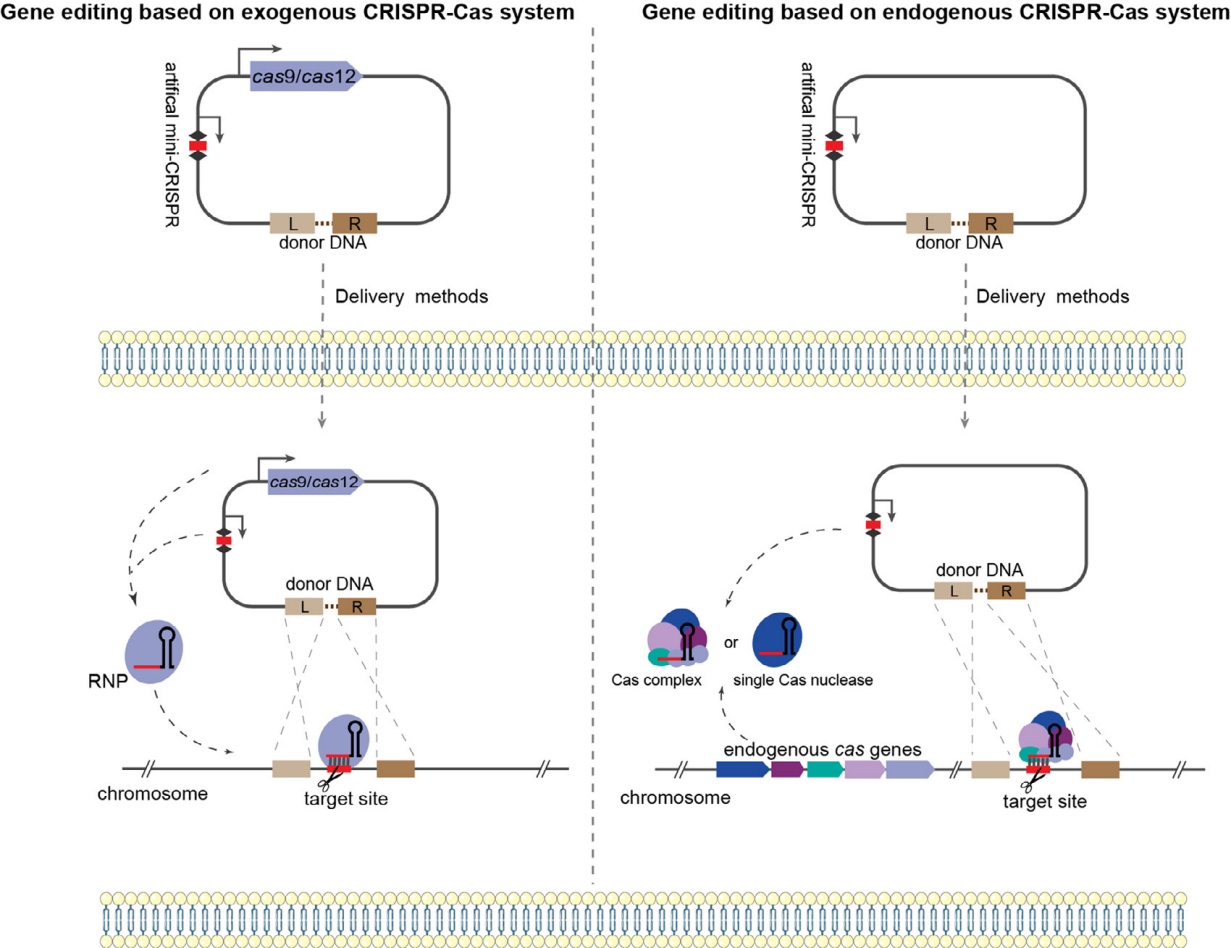
Strategies for gene editing based on exogenous and endogenous CRISPR-Cas systems. For gene editing based on exogenous CRISPR-Cas systems, Cas nuclease (usually Cas9 or Cas12) and short artificial mini-CRISPR arrays are provided to create double-stranded breaks at specific sites. Donor DNA is also needed for the introduction of desired mutations via the HDR pathway. For gene editing based on endogenous CRISPR-Cas systems, only artificial mini-CRISPR arrays and donor DNA are needed. The crRNA generated by artificial mini-CRISPR arrays forms effector complexes with endogenous Cas proteins, resulting in cleavage of target sites. Subsequently, the target mutation is achieved by homologous recombination between the donor DNA and host genome.

Two general strategies are used to induce DSBs at specific loci in the genome through CRISPR systems: one is to introduce a complete set of exogenous CRISPR-Cas systems into the host, while the other is to directly use the CRISPR-Cas system encoded by certain prokaryotes for gene editing (Fig. 4). In gene editing using exogenous CRISPR-Cas systems, class 2 CRISPR-Cas systems are often preferred. Because of the simplicity of class 2 systems, only a single Cas protein is needed to complete the identification and cleavage of target sites, which facilitates their modification and exploitation. Among these, the type II CRISPR-Cas9 system was the first identified class 2 system and was also the first to be used in gene editing. In the past few years, the CRISPR-Cas9 system has been widely used for genome editing in a variety of organisms [13,14]. The first developed and applied Cas9 protein was SpCas9 from *Streptococcus pyogenes*, which has strong cleavage activity in cells, especially in eukaryotic cells [33]. However, the need for wild-type SpCas9 for specific PAM sequences limits its application, especially when performing precise genome editing, such as single base editing [28,33]. To address this problem, many Cas9 homologs and variants with broad PAM preferences have been mined and characterized for gene editing using directed evolution or bioinformatics [71,72]. Following CRISPR-Cas9, the CRISPR-Cas12 system has been developed and applied. It has the advantages of a simple structure, wide application range, and high genome editing efficiency [67,68]. The development of Cas12 systems has further enriched the library of available PAM sequences. It is easier to use Cas12a (also known as Cpf1) as an exogenous CRISPR-Cas system because of its ability to process crRNA autonomously without the involvement of other helper proteins or trans-activating crRNAs (tracrRNAs) [15]. The ease of crRNA processing also gives the Cas12a system great significance for multiplex gene editing [15]. Additionally, some Cas12a variants with a lower preference for PAM sequences and a stronger target specificity are also gradually being developed [28,73]. The editing activity of Cas12a can also be enhanced by modifying the crRNA structure [74]. In general, there are two main points of gene editing with the exogenous CRISPR-Cas system. These include methods to characterize the Cas nucleases that work properly in target host cells and strategies for delivering editing elements, such as CRISPR arrays, large size heterologous nucleases, and donor DNA for damage repair, into cells.

Undoubtedly, gene editing strategies based on exogenous class 2 systems like CRISPR-Cas9/Cas12a have attracted much attention because of their fruitful results with genome manipulation. However, this strategy may be more suitable for cells that do not encode their own CRISPR systems. Furthermore, gene editing tools based on exogenous CRISPR systems still have significant limitations in some cases. First, the complex intracellular environment and growth conditions of extremophiles may affect the activity of the commonly used Cas nucleases. In addition, the large size of heterologous Cas nucleases may prevent their introduction into some organisms because of their intrinsic proteotoxicity [75,76]. Additionally, given the wide distribution of CRISPR-Cas systems in prokaryotes (in ∼50% of bacteria and ∼90% of archaea) [32,33], a better strategy for gene editing in prokaryotes is to repurpose their endogenous CRISPR-Cas systems. In contrast to the introduction of exogenous class 2 systems, all components of the endogenous CRISPR-Cas system are present in the cell, which avoids the toxicity associated with heterologous nucleases. The maturation of crRNA also relies exclusively on endogenous processing proteins, without the need to provide any additional exogenous proteins. Only an artificial CRISPR array and homologous DNA template for damage repair are needed to achieve precise editing of target sites, which is particularly convenient for simultaneous multi-gene editing and regulation of metabolic engineering [77]. Currently, different types of endogenous CRISPR-Cas system-mediated gene editing in prokaryotes are quite well established and are widely used in many bacteria and archaea, including extremophiles [77], [78], [79]. For example, the endogenous I-A and III-B systems have been successfully used for genome editing of the thermophilic archaeon *Sulfolobus islandicus* [78]. Notably, for the *Zymomonas mobilis* strain, which is valuable for ethanol production, the exogenous Cas9 system can only mediate very inefficient gene editing. Recent studies have enabled efficient gene editing by characterizing and repurposing its endogenous I-F type CRISPR-Cas system [80]. In addition, both the type II-A system of the lactic acid-producing bacterium *Pediococcus acidilactici* and the type I-B system of the halophilic archaea *Haloarcula hispanica* all demonstrated high editing efficiency when performing genome editing [81], [82], [83]. Furthermore, our recent study showed that several different types of endogenous CRISPR-Cas systems (including types IIIA/B, I-B, and I-C) encoded by *Thermus thermophilus*, a model strain for studying thermophiles, can be used for their genome editing, all with high editing efficiency [84]. Multiple different types of CRISPR-Cas systems being simultaneously encoded is relatively common in prokaryotes, especially in archaea [32,33]. The identification and utilization of their different endogenous CRISPR-Cas systems can also break through the limitations of a single system for PAM sequences. With the rapid development of sequencing, as well as bioinformatics technologies, endogenous CRISPR-Cas systems will be able to be rapidly characterized from specific genomes. This will also facilitate the development of endogenous CRISPR-Cas systems as the next generation genome editing technology for prokaryotes.

### 4.2 CRISPR-based gene editing independent of DSBs

**Fig. 5:**
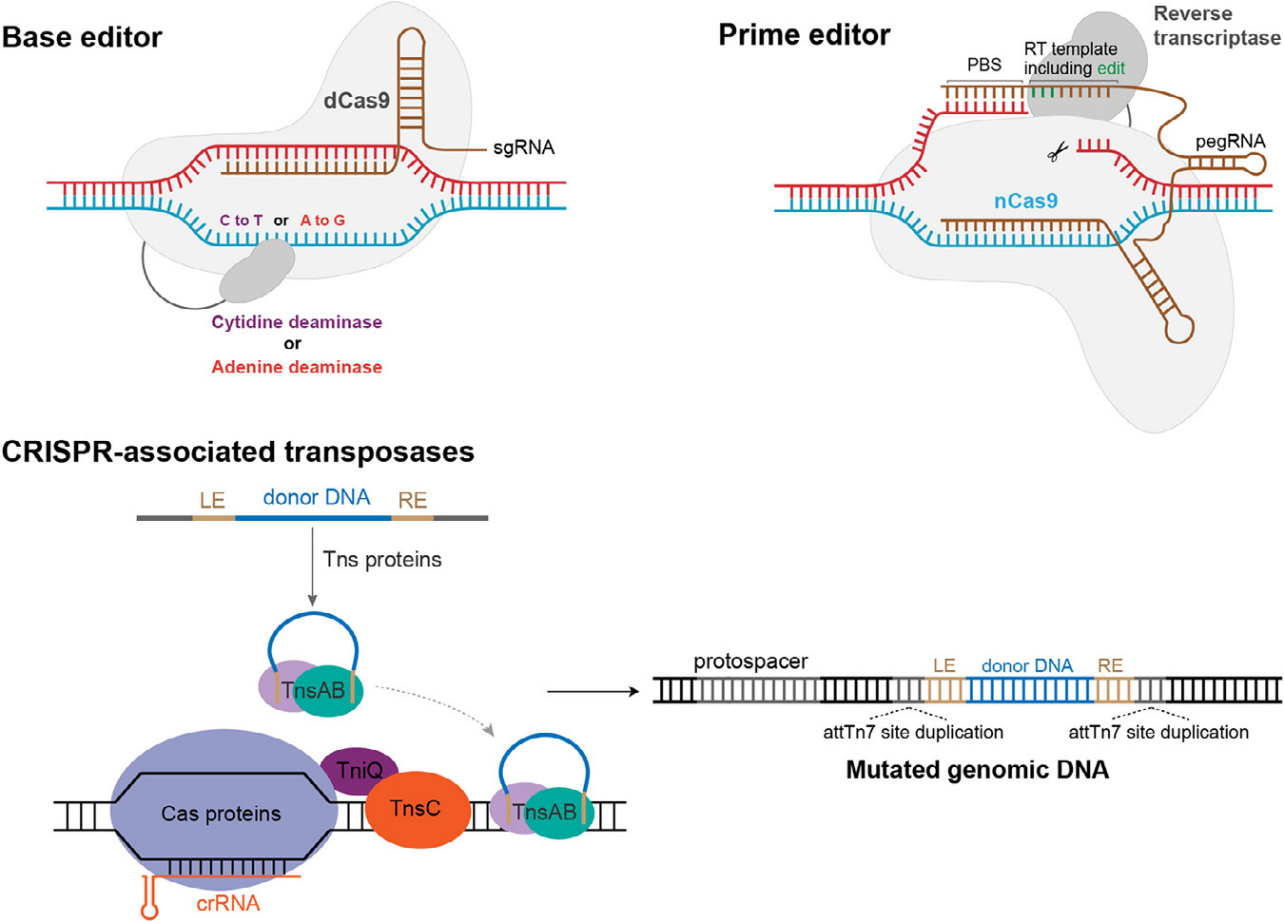
CRISPR-based gene editing tools without DSBs: base editors (BEs), prime editors (PEs), and CRISPR-associated transposases. For BEs, dCas9 or nCas9 is fused with deaminase. When the sgRNA guides Cas9 to bind to the target, deaminase deamidates the unwound single-stranded DNA, ultimately resulting in base conversion. For PEs, nCas9 is fused with reverse transcriptase and the sgRNA was engineered to a more complex pegRNA. Following the actions of nCas9, the 3′ end of the pegRNA can pair with the broken single-stranded DNA and designed mutations are then introduced into the target site under the action of reverse transcriptase. For CRISPR-related transposase (CAST)-mediated gene integration, transposon donor DNA is first captured and processed by TnsA and TnsB. Then, TnsA, TnsB, and the processed substrate form an integrated complex. The CRISPR elements of CAST systems bind to a specific site under the guidance of crRNA and recruit TniQ, TnsC, and TnsA/B complexes to integrate the donor DNA fragment downstream of the target site.

Early gene editing techniques based on ZFNs, TALENs, and CRISPR-Cas systems all rely on the induction of DNA DSBs at target sites, which in turn activate host DNA damage repair pathways, including MMEJ, NHEJ, and HDR. Among these repair pathways, MMEJ and NHEJ can easily cause random insertions and deletions that are very detrimental to the host. Although HDR can introduce accurate mutations of interest according to the donor DNA we provide, the efficiency of homologous recombinant repair in cells is relatively low (especially for eukaryotes, ∼0.1% to 5%) [85]. In addition, DSBs caused by potential off-target effects of CRISPR systems may also be quite harmful. Therefore, the introduction of DSBs has certain risks to the host. To address this problem, the CRISPR-Cas system has been further developed to obtain BE and PE technologies. In addition, the establishment of CASTs technology has also further expanded the application scope of gene editing tools that do not rely on DSBs (Fig. 5).

#### 4.2.1 BEs

The novel gene editing technology, BEs, that allows single-base conversion without DNA DSBs or homologous templates was first proposed by David R. Liu's team in 2016 [19]. This technology is based on the CRISPR-Cas9 system, using dead Cas9 (dCas9) or Cas9 nickase (nCas9) that do not have intact nuclease activity. Cas9 possesses two nuclease domains, HNH and RuvC, which are responsible for cleaving the target and non-target strands, respectively. Both nuclease domains of dCas9 are mutated, thus its DNA cleavage activity is abolished. However, its ability to bind to target DNA is still preserved. In contrast, only one nuclease domain is mutated in nCas9, so just one DNA strand can be cleaved [86]. In addition to using dCas9 or nCas9, the core of BEs is the fusion of deaminase with dCas9/nCas9. The introduction of cytosine deaminase and adenine deaminase yielded cytosine base editors (CBEs) and adenine base editors (ABEs), which enabled the conversion of C-G to T-C and A-T to G-C, respectively [19,87]. For CBEs, cytosine deaminase deamidates cytosine (C) in exposed ssDNA to uracil (U), and the U-G mismatch is subsequently repaired by the host to U-A, eventually forming a T-A base pair. In addition, the excision of uracil was inhibited by the introduction of a uracil glycosylase inhibitor (UGI), which further improved the editing efficiency of CBEs [28]. For ABEs, adenine (A) is converted to inosine (I) by the action of adenine deaminase, and inosine is misidentified by cells as guanine (G) [87]. Subsequent studies have focused on improving the efficiency, specificity, and transition range of BEs. The reported optimization strategies mainly include the following: 1) using engineered deaminases with higher activity and specificity [28], 2) using single-stranded binding proteins to promote R-loop formation and prolong the exposure time of ssDNA substrates [88], 3) modifying deaminases to avoid inhibition of their activity by some epistatic modifications [28], 4) optimizing linker sequences to improve deaminase stability and activity [28], 5) increasing the copy number of UGI in CBEs to reduce the production of insertions and deletions [28], 6) using engineered deaminases with lower affinity for ssDNA or RNA to reduce off-target effects [22], 7) altering the editing site by changing the position of the deaminase fusion [28,89], 8) improving the specificity and reducing the PAM preference by directed evolution of Cas9 [90,91], and 9) fusing cytosine deaminase with adenine deaminase to nCas9 to obtain a Two-base editor [92]. In addition, some recent studies have reported a wider range of BEs, such as CGBE (C to G), GBE (C to A), and AYBE (A to C/T) [93], [94], [95], [96], [97]. However, multi-base mutations, insertions, and deletions of DNA fragments are still incomplete, and BEs require further optimization to reduce their off-target effects.

#### 4.2.2 PEs

To overcome the limitations of BEs, PEs were proposed by David R. Liu's team in 2019 [98]. This system enables various types of base substitutions and precise DNA insertions and deletions without relying on DSBs and exogenous template DNA. Cas9 nickase (H840A) is expressed in fusion with reverse transcriptase (RT), and this fusion protein is then guided by a prime editing guide RNA (pegRNA) to achieve gene editing at the target site. The pegRNA is mainly a modification to the 3′ end of the single guide RNA (sgRNA). The pegRNA is composed of three main parts: the sgRNA portion at the 5′ end, a hairpin structure at the 3′ end of the sgRNA followed by the addition of a template for reverse transcription (RT template, RTT), and a primer binding site (PBS) for hybridization with the 3′ end of the nicked DNA strand to initiate reverse transcription. Guided by the pegRNA, nCas9 cuts the PAM-containing target DNA strand and the broken strand is base-paired to the PBS sequence at the 3′ end of the pegRNA. This is followed by a reverse transcription reaction using the RTT sequence as a template for RT. The 3′ DNA flap formed by reverse transcription displaces the 5′ DNA flap on the target DNA strand, eventually introducing the desired mutation into the target site after the 5′ DNA flap is cleaved and the genome is repaired [98]. Theoretically, we can easily achieve various edits using PEs. However, the gene editing efficiency of PEs in practical applications is still quite low. How to enhance the PE editing efficiency has become a pressing issue, and several methods have been reported to improve their efficiency, including optimizing pegRNAs to improve their transcription levels and stability [99,100], using more active engineered RTs and nCas9 [101,102], and manipulating the host repair pathway [25,103]. Although PEs are capable of more flexible gene editing, the current efficiency of PE-mediated editing remains much lower than that of BEs [100]. In addition, the delivery vehicle for BEs and PEs is also a major hindrance limiting their application because of the large fusion proteins.

#### 4.2.3 CASTs

In addition to BEs and PEs, the CAST system has also been developed as a gene editing tool that does not rely on DSBs. While exploring the diversity and evolution of the CRISPR-Cas system, it was recently discovered that some prokaryotic CRISPR elements were hijacked by the Tn7 transposon, and this co-evolution resulted in the CAST system [104,105]. CAST systems can mediate DNA transposition under the guidance of crRNA, so they can be deployed as a programmable gene integration tool [106,107]. In CAST systems, CRISPR nucleases are often inactive. For example, some type I systems lack the important Cas3 helicase-nuclease domain and certain type V systems are predicted to have no nuclease activity [104,108]. This CRISPR-Cas system, which lacks nuclease activity, acts as a genomic targeting module for CAST systems. A better understanding of the composition and function of the Tn7 transposon will help further develop this new tool. Tn7-like transposons typically contain the *tnsA, tnsB, tnsC, tnsD*, and *tnsE* genes, as well as donor DNA embedded between left end/right end (LE/RE) sequences. Typically, TnsB recognizes the transposon end sequence and binds to TnsA, which processes the 3′ and 5′ ends of substrate, respectively. TnsA, TnsB, and the processed donor then form an integrated complex (some systems lack TnsA) [109]. TnsD/E is a sequence-specific DNA-binding protein responsible for target selection. Communication between TnsD/E and TnsA/B complexes is mediated by the ATP hydrolyzed protein TnsC. TnsC interacts with TnsD/E to coordinate transposase recruitment and insert donor sequences into conserved Tn7 sites [109,110]. The CAST system replaces TnsD/E with the associated Cas protein and TniQ, a homolog of TnsD, for DNA targeting. Briefly, the CRISPR elements of CAST systems (usually Cas12k or Cascade) bind to a specific site under the guidance of the crRNA and recruit TniQ, TnsC, and TnsA/B complexes to integrate the donor DNA fragment downstream of the target site. The entire process of CAST system-mediated gene integration does not produce DSBs and gene editing technologies developed based on CASTs can therefore overcome the efficiency limitations associated with homologous recombination and the negative effects caused by DSBs. CASTs have been deployed in a variety of prokaryotes and have shown great potential for multiple integration of large fragments and targeted editing in microbial communities [111], [112], [113].

## 5 Application of CRISPR-based gene editing in engineering microbiology

Microorganisms have a long application history in different fields, such as agriculture, industry, medicine, and food. They are at the core of modern industrial biotechnology, and their importance to human life is becoming increasingly evident. With the development and application of modern biological techniques, researchers are gradually transitioning from simply isolating strains from nature to artificially constructing superior strains using gene editing techniques. Excellent microbial strains are important for improving the quality and yield of fermentation industrial products. Additionally, efficient breeding techniques are crucial for the application of microbial resources. CRISPR-based gene editing technology, a hot technology in modern genetics research, has greatly improved the efficiency of modern industrial microbial breeding. This section focuses on the application of gene editing technology based on the CRISPR-Cas system to industrial microorganisms and probiotic modifications.

### 5.1 Application to industrial microorganisms

Excellent chassis microbial cells are of great importance for bioindustrial production. Gene editing technology allows for targeted genetic improvement of strains. Furthermore, the development of the CRISPR-Cas system has provided a very simple and efficient gene editing tool for the modification and improvement of industrial chassis cells. By artificially designing the metabolic network of microorganisms, optimizing the suitability of components and chassis, and achieving fine regulation of metabolic flow, we can establish a cell factory that is close to the ideal state. This can help achieve target product production with optimal quality and quantity. Using genetic engineering techniques and means, the microorganisms involved in this production can be artificially modified to produce compounds that have not yet been synthesized or are difficult to manufacture. Additionally, by discovering and optimizing chassis microorganisms, new and more efficient industrial processes can be achieved to produce purer and cheaper products. Currently, the main industrial chassis microorganisms that are commonly used as cell factories include *Escherichia coli, Corynebacterium glutamicum, Bacillus subtilis*, and *Saccharomyces cerevisiae*. Among them, *E. coli* is widely used in industrial fermentation because of its ease of cultivation, short proliferation cycle, and high tolerance to the environment. In 2013, Jiang et al. first validated the ability of the CRISPR/Cas9 system to target cleavage of the genome in *E. coli* and were able to introduce mutations into the host through repair of λ-Red proteins. This confirmed the ability of the system to combine recombinant systems for effective gene editing in *E. coli* with a mutation rate of 65% [114]. Since then, CRISPR-Cas9-based gene editing technology has been widely used to improve the production performance of *E. coli*. Li et al. developed a CRISPR-Cas9-based method for iterative editing and metabolic engineering of *E. coli*. This method has allowed us to introduce various types of genomic modifications with nearly 100% editing efficiency. They also used this platform to optimize the β-carotene production-related pathways in *E. coli* and constructed a library of over 100 mutations. The final screened strain containing 15 mutant genes achieved a β-carotene yield of 2.0 g/L [115]. *E. coli* Nissle 1917 is an endotoxin-free probiotic for the treatment of gastrointestinal disorders. Lan et al. successfully eliminated two cryptic plasmids of *E. coli* Nissle 1917 by CRISPR-Cas9-based plasmid curing, reducing its metabolic burden and ultimately producing gamma-aminobutyric acid (17.9 g/L) with an antibiotic-free system [116]. This paved the way for efficient engineering of *E. coli* Nissle 1917 as live therapeutics. Genistin is a biologically active isoflavone glycoside of great nutritional and pharmaceutical significance. At present, isoflavones on the market are produced by direct extraction from plants. However, its low abundance in plants and structural complexity hinder the acquisition of this botanical medicine through plant extraction or chemical synthesis. Wang et al. artificially constructed a pathway for genistin synthesis using glycerol in *E. coli*, significantly increasing the genistin yield through CRISPRi-mediated metabolic regulation. As a result, the optimized strain was able to produce up to 202.7 mg/L of genistin from glycerol in batch fermentations [117]. This study represents the first report of genistin biosynthesis from glycerol, laying the groundwork to produce glucoside isoflavones using low-cost microorganisms. More importantly, it may provide a reference for the biosynthetic pathway engineering of other microbial hosts to achieve green production of complex natural products. In addition, improving the stress resistance of chassis microorganisms is also very important for industrial production. Seo et al. used the CRISPR-Cas9 system to integrate acid tolerance genes (*dsrA* and *rcsB*) into the genome of *E. coli* BL21 and significantly improved its tolerance to n-heptanoic acid [118]. Ou et al. successfully achieved normal growth of BL21(DE3) in the presence of T7 phage at a concentration of up to 2 × 10 [7] PFU/mL by deploying the CRISPR-Cas9 defense system to the strain's genome [119]. *C. glutamicum* is a Gram-positive soil bacterium that was commonly used in the industrial production of amino acids and has also been adapted to synthesize various compounds, including pharmaceutical intermediates and biofuels [120]. Currently, the developed high-efficiency gene editing tools for *C. glutamicum* are mainly based on the CRISPR-Cas9 and CRISPR-Cpf1 systems. In addition, the use of CBE and ABE in *C. glutamicum* has also been reported [121]. Zhao et al. were able to delete large segments and simultaneously perform multi-gene editing of *C. glutamicum* with high efficiency by combining the CRISPR-Cpf1 and RecE/T systems [122]. This study was the first to use the Cpf1 system to achieve effective multiplex gene editing and large fragment deletions in *C. glutamicum*, which provides an efficient and simple tool for its genome editing to further accelerate metabolic engineering and genome evolution. Park et al. used the CRISPRi method to control the metabolic fluxes of the L-lysine and heterologous squalene synthesis pathways in *C. glutamicum*, in which pyruvate, an intermediate in the central metabolism, was regulated by repressing the gene encoding pyruvate carboxylase. As a result, the first efficient simultaneous synthesis of L-lysine and squalene in *C. glutamicum* was achieved [123]. Moreover, this co-production strategy provided a reference for the synergistic production of amino acids and multiple terpenes using *C. glutamicum*. Li et al. identified that the *gltA* gene, which is involved in the TCA cycle, is a key gene in the O-Acetylhomoserine (OAH) biosynthetic pathway in *C. glutamicum* using the CRISPR-Cas9 system, facilitating the industrial production of OAH from *C. glutamicum* [124]. Although the production of OAH by Corynebacterium glutamate is relatively mature, some limiting factors have been successfully identified by using CRISPR technology, which has further improved the production efficiency of OAH and has significant application value.

*B. subtilis* is a model strain of Gram-positive *Bacillus* with non-pathogenicity, strong extracellular protein secretion ability, and no significant codon preference. It is a GRAS (generally recognized as safe) grade food safety strain with great potential for the production of nutraceuticals, chemical products, and enzyme preparations [125]. Gene editing and regulatory tools based on the CRISPR-Cas system have also been developed for *B. subtilis*. Altenbuchner et al. and Wu et al. used the CRISPR-Cas9 and CRISPR-Cpf1 systems to achieve long fragment gene deletion and multi-point mutation of *B. subtilis*, respectively [126,127]. Recently, Zou et al. also achieved efficient iterative gene editing of *B. subtilis* using an all-in-one plasmid-based CRISPR-Cas9 system with near 100% efficiency for different types of mutations [128]. The development of these efficient CRISPR-based gene editing tools for *B. subtilis* has greatly promoted the modification of *B. subtilis* chassis cells and accelerated its industrialization. Fehler et al. also used the CRISPR-dCas9 system to knock down the transcript levels of flagellar-associated genes in *B. subtilis*, resulting in a significant 2- to 3-fold increase in α-amylase production [129]. In addition, as a eukaryotic model organism commonly used in industrial production, *S. cerevisiae* is often used as a host cell in the synthetic biology and metabolic engineering fields to produce a variety of high value-added chemicals because of its clear and simple genetic background, ease of operation, and safety [130]. To meet the needs of scientific research and industrial production, gene editing technology based on the CRISPR-Cas system is also becoming increasingly sophisticated in *S. cerevisiae*. The gRNA-tRNA array-mediated CRISPR-Cas9 (GTR-CRISPR) system developed by Zhang et al. for multiple gene editing in *S. cerevisiae*, which was used to successfully simultaneously knock out eight genes with 80% efficiency, greatly improved the editing efficiency in this species [131]. The lipid metabolic network of *S. cerevisiae* was simplified by using this GTR-CRISPR system, and its free fatty acid production was increased by 30-fold [131]. In addition, the biosynthesis process of bioethanol, artemisinin, and terpenoids in *S. cerevisiae* was optimized by CRISPR-Cas9 technology, with the yields of these substances being greatly enhanced [132,133]. These and the many cases shown in Table 2 demonstrate that the applications of CRISPR-Cas-based gene editing or regulation technologies in industrial chassis microorganisms’ improvement have been very extensive. As new efficient, innovative, and practical CRISPR-Cas technologies continue to be developed, there will be further advancements in the research and development of these industrial chassis microorganisms.

**Table 2:** Applications of CRISPR-based gene editing in industrial chassis microorganisms.

Strains	Editing system	Productions	Description	Ref.
*E. coli*	exogenous Cas9	-	First application in *E. coli* genome editing	[114]
	exogenous Cas9	β-carotene	2.0 g/L	[115]
	exogenous Cas9	gamma-aminobutyric acid	17.9 g/L	[116]
	exogenous dCas9	genistin	202.7 mg/L	[117]
	exogenous Cas9	heptanoic	improved tolerance to heptanoic	[118]
	exogenous Cas9	-	resistance to T7 phage	[119]
*C. glutamicum*	exogenous Cpf1-RecE/T	-	multiplex gene editing and large DNA fragment deletion	[122]
	exogenous dCas9	l-lysine, squalene	co-production of l-lysine and heterologous squalene	[123]
	exogenous dCas9	lysine	efficient multiplex gene repression; increased the lysine titer and yield for over 4.0-fold	[134]
	exogenous Cas9	O-Acetylhomoserine	identified key gene in the O-Acetylhomoserine biosynthetic pathway	[124]
*B. subtilis*	exogenous Cas9	-	large fragment deletion	[126]
	exogenous Cas9	α-amylase	the α-amylase activity in the absence of added xylose was 1.2-fold greater	[135]
	exogenous Cpf1	-	multiple genes editing and regulation	[127]
	exogenous Cpf1	hyaluronic acid	1.39 g/L	[136]
	exogenous Cas9	-	efficient iterative gene editing	[128]
	exogenous dCas9	α-amylase	improvement in yield by 2-3 folds	[129]
*S. cerevisiae*	gRNA-tRNA mediated exogenous Cas9	free fatty acid	improvement in yield by nearly 30 folds	[131]
	exogenous Cas9	ethanol	improved ethanol tolerance and production	[130]
	exogenous Cpf1	D-lactic acid	improvement in productivity and yield by 2.2 and 1.5 folds	[137]
	exogenous Cas9	artemisinin	740 mg/L	[138]
	exogenous Cas9	β-carotene	improvement in yield by 3 folds	[139]

### 5.2 Application to probiotics

Probiotics are defined as live microorganisms that, if given in sufficient quantities, are beneficial to host health [140]. Probiotics have been used in clinical and food fields for many years, and their safety and effectiveness have gradually gained recognition. For their specific active ingredients and health promotion mechanisms, most of the current studies have focused on their effects on the intestinal microbiome. Existing studies have shown that probiotics can promote intestinal health in several ways, including by improving the structure of the intestinal microflora, inhibiting the growth of pathogens, and participating in the immune response [141]. In particular, strains from *Lactobacillus* and *Bifidobacterium* represent the two main groups commonly used for probiotic development and food addition. There are also probiotics from other genera, such as *Lactococcus lactis, Streptococcus thermophilus*, and *Bacillus coagulans* [142]. These strains are generally capable of fermenting dietary fiber into short-chain fatty acids (e.g., acetate and butyric acid) in the intestine to provide energy to the host. They can also play an important role in vitamin synthesis [143]. With the continuous popularization of genome sequencing technology and the development of microbial genome editing tools based on the CRISPR-Cas system, researchers have gradually shifted their focus from isolating probiotics from the natural environment to modifying probiotics to develop customized ones [144]. Through probiotic gene editing, we can introduce target genes with positive effects and optimize and reconstruct metabolic pathways to increase the yield of target metabolites. However, because most probiotics still lack a stable genetic transformation system, the popular CRISPR-based gene editing technology has only been established in a few probiotics. Most still remain in studies, such as gene knockout and regulation, with fewer reports of large segments of exogenous functional genes knocked in and stably expressed, and even fewer animal experiments. Specifically, the exogenous CRISPR-Cas9 system was used for the first time as a screening tool to assist in ssDNA recombination, achieving a codon saturation mutation in *Lactobacillus reuteri* [145]. Subsequently, Cas9 nickase-based gene editing tools were also successfully developed in *Lactobacillus casei*, which uses nCas9 to generate ssDNA breaks on chromosomes to trigger the HDR process and achieve precise manipulation of the genome [146]. More recent studies have shown that the CRISPR-nCas9 system enables efficient gene editing in a variety of *Lactobacillus* species, including *Lactobacillus acidophilus, Lactobacillus gasseri*, and *Lactobacillus paracasei* [147]. Tian et al. obtained an engineered *Lactobacillus paracasei* strain capable of efficient production of high-purity L-lactic acid by combining CRISPR-Cas9 gene editing technology with an adaptive evolutionary strategy [148]. Overall, these versatile exogenous Cas9-based systems facilitate genome editing of probiotics and represent a valuable way to perform gene editing in species that do not possess an endogenous CRISPR-Cas system.

In addition, it has been shown that about 60% of the 1,262 publicly available *Lactobacillus* genomes can be detected with CRISPR arrays, and about 40% have intact CRISPR-Cas systems [149]. This indicates that there is a relatively wide distribution of CRISPR-Cas systems in *Lactobacillus*. As more genome sequences of probiotics are resolved, their encoded endogenous CRISPR-Cas systems are gradually characterized, including *S. thermophilus, Lactobacillus sakei, Pediococcus acidilactici*, and others [82,[150], [151], [152]]. There are already cases of genomic manipulation of various probiotic bacteria using the endogenous CRISPR-Cas system. For *Lactobacillus crispatus*, where plasmid transformation and gene editing have been previously difficult to achieve, its endogenous type I-E CRISPR-Cas system has been used for efficient gene editing [153]. Liu et al. identified the type II-A CRISPR-Cas system of *Pediococcus acidilactici* and developed it into an efficient gene editing tool. This also improved the growth performance and lactate production of *P. acidilactici* [82]. *Bifidobacterium* is the second most commonly used genus in commercial probiotic products (after *Lactobacillus*) and is an important intestinal commensal. Researchers analyzed 954 public *Bifidobacterium* genomes, identified CRISPR-Cas systems in 57% of these strains, and further characterized the crRNA and PAM sequences. This provided a molecular basis for the development of new genome editing tools for various applications [152]. In a recent study, both the endogenous type I-G system of *Bifidobacterium* and exogenous CBE could be used for genome engineering of *Bifidobacterium lactis* [154]. In addition to genetic modification of existing probiotics, researchers have recently constructed engineered probiotics using *E. coli* Nissle 1917 as a host bacterium. This artificially customized probiotic can inhibit *C. difficile* infection in mice, providing a new direction for developing engineered probiotics [155]. Additional examples of gene editing of probiotics are listed in Table 3. Typical traditional probiotics (mainly *Lactobacillus* and *Bifidobacterium*) are currently being developed as in vivo diagnostics and therapeutics to improve human health. However, their specific probiotic basis is still not fully understood, which may slow down the translation process in the functional food and pharmaceutical fields. The development of efficient gene editing tools based on the CRISPR-Cas system will facilitate a better understanding of the physiological properties of probiotics and molecular mechanisms underlying their interactions with hosts and host microbial communities. This will therefore further facilitate the development of next generation engineered probiotics with higher activity and customized functions.

**Table 3:** Applications of CRISPR-based gene editing in probiotics.

Probiotics	Editing systems	Descriptions	References
*L. reuteri*	exogenous Cas9	CRISPR–Cas9 selection combined with ssDNA recombineering; successfully applied codon saturation mutagenesis in the *L. reuteri* chromosome.	[145]
*L. casei*	exogenous Cas9 nickase (D10A)	efficient single-gene deletion and insertion (25 to 62%).	[146]
*L. plantarum*	exogenous Cas9	recombination efficiency was effectively improved; *L. plantarum* WCFS1 produced 797.3 mg/liter N-acetylglucosamine without introducing exogenous genes or plasmids.	[156]
*Lactococcus lactis*	exogenous dCas9	dCas9 enabled CRISPR interference-mediated silencing of single or multiple target genes with significant reduction of gene expression, up to 99%.	[157]
*L. acidophilus*	exogenous Cas9 nickase (D10A)	deletions ranging between 300 bp and 1.9 kb at various loci, yielding 35 to 100% mutant recovery rates.	[147]
*L. gasseri*	exogenous Cas9 nickase (D10A)	concurrent single-base substitution and gene deletion.	[147]
*L. paracasei*	exogenous Cas9 nickase (D10A)	in-frame gene deletion.	[147]
*L. paracasei*	exogenous Cas9	an engineered strain producing high optical purity L-lactic acid was constructed.	[148]
*L. crispatus*	endogenous type I-E	a 643-bp deletion (100% efficiency), a stop codon insertion (36%), and a single nucleotide substitution (19%).	[153]
*P. acidilactici*	endogenous type II-A	high-efficiency markerless gene deletion, gene integration, and point mutation; enhanced both cell growth and lactic acid production.	[82]
*B. lactis*	endogenous type I-G	screened for naturally occurring large deletions up to 27 kb and generated a 500-bp deletion in *tetW.*	[154]
*B. lactis*	exogenous CBE	installed C•G-to-T•A amber mutations to resensitize multiple B. lactis strains to tetracycline.	[154]

## 6 Conclusions and prospects

The development of new research tools and technologies is undoubtedly essential for advancing science. The exploitation of CRISPR-Cas systems derived from bacteria and archaea has been one of the most rewarding research achievements of the last decade. CRISPR-based biotechnology has provided researchers with an unprecedented powerful toolbox. Currently, various types of Cas nucleases and their engineered variants have been used in various aspects of scientific research, as well as for industrial production. The development of gene editing tools based on the CRISPR-Cas system has also been very rapid, with a series of breakthrough new technologies emerging, such as BEs, Pes, and CASTs. This has further broadened their scope of use.

The CRISPR-Cas9 and CRISPR-Cpf1 systems are widely used in gene editing because they use single-protein RNP complexes for target cleavage and are the most well-known systems. Although gene editing tools based on these two systems have demonstrated efficient gene manipulation in numerous eukaryotic and prokaryotic organisms [13], [14], [15], there are still several outstanding issues that need to be addressed. Firstly, heterologous Cas9 and Cpf1 proteins serving as a nuclease may be cytotoxic to the host [75,76]. Moreover, the differences in toxicity of the various Cas nucleases in different organisms have not been well explained. As the ranks of industrial microbial chassis cells continue to grow, the universal use of Cas nucleases will be further severely challenged. Secondly, how to efficiently deliver large-sized Cas9 and Cpf1 proteins into host cells is also a matter to be considered. For gene editing in prokaryotes, the use of the endogenous CRISPR-Cas system for gene editing can alleviate these problems to some extent. However, for different bacteria and archaea, their encoded CRISPR-Cas systems may have large differences. This may make the identification of PAM sequences, Cas protein activity, and crRNA characteristics a tedious process. How to combine these two different strategies scientifically and apply them in different organisms will be an important research direction. For the modification of industrial chassis microorganisms and probiotics, gene editing cases based on endogenous and exogenous CRISPR-Cas systems both exist (Tables 2 and 3). Recently, Zhang's team reported a programmable protein delivery system that uses a virulent injection system derived from bacteria [158]. This delivery system can overcome many delivery barriers, such as host cell walls, cell membranes, and the cytosol. In addition, it has the ability to deliver both small proteins and larger proteins, like Cas9 [158], which is expected to be a universal protein delivery platform. For common industrial chassis microorganisms, gene editing methods based on different CRISPR-Cas systems are relatively mature. Using these systems for metabolic engineering to make them more suitable for industrial production is a future direction. It is also important to use these gene editing methods to develop novel industrial chassis microorganisms. For the genetic engineering of probiotics, more basic work must be performed, including the establishment of plasmid transformation systems and characterization of endogenous CRISPR-Cas systems. It is worth emphasizing that there are several notable common issues that require attention, regardless of the type of CRISPR-Cas system employed, endogenous, or exogenous Cas nuclease strategy. Firstly, the CRISPR-Cas system has a PAM preference, which limits the editing of arbitrary loci. Although some engineered Cas nucleases with low dependence on the PAM sequence have been developed by means of directed evolution [72,159,160], the site-dependence has not been completely resolved. Therefore, improving the pervasiveness of CRISPR technology relies on establishing a method that does not require PAM sequences or by rational design to recognize base sequences in highly conserved regions of genes, such as TATA or NTG. Current studies have identified Cas14 proteins that do not require PAM sequences when targeting DNA [161], and Cas14 thus has the potential to address the dependence of CRISPR technology on PAM sequences. Secondly, the off-target effects of the CRISPR-Cas system is also worth our attention, and the current solution strategy mainly relies on optimization of the sgRNA [162], [163], [164] and modification of the Cas protein [165], [166], [167]. Additionally, techniques for efficient detection of off-target events still need to be developed. The development of technologies for precise and timely shutdown of the CRISPR-Cas system activity is also crucial, as this can further reduce the negative impact of these exogenous elements on the host. In recent years, the discovery of the Anti-CRISPR (Acr) protein has held promise for precise control of the CRISPR-Cas system [31,168]. In addition, an understanding of the mechanism of escaping the CRISPR-Cas system is also important, which will further improve the efficiency of gene editing. Notably, although CRISPR-based genome manipulation technologies have been very diverse, their application potential in the industrial field needs to be further explored. Some emerging CRSPR-based tools have mostly been applied in eukaryotes, and there are few studies in the field of microbial-related industrial fields, such as BEs, PEs, CASTs, and RNA editing technologies. The reverse transcriptase and RNA editing catalase required for genome editing in industrial organisms, such as *S. cerevisiae*, still need to be further explored. In addition, the development of multiple orthologous systems can regulate the expression of different genes in various ways, which is also very important for gene network optimization. Along with the expansion of industrial chassis organisms, the mining of new genome editing technologies is also a focus of industrial biotechnology research. Further optimization and innovation are needed for the current technologies with low efficiency and specificity. With the continuous progression of bioinformatics methods and the rapid development of artificial intelligence technology, genome editing approaches may be developed based on this new model of artificial intelligence. Although some issues have yet to be completely resolved, they will drive the continued development of CRISPR-based gene editing technology. We believe that the CRISPR-Cas system will be further refined in the future, and the toolbox for genome engineering research will continue to be expanded by combining it with new functional components. The development of industrial biotechnology will also usher in an explosive period.

## Declaration of Competing Interest

The authors declare that they have no known competing financial interests or personal relationships that could have appeared to influence the work reported in this paper.
